# Physical activity, sedentary behavior, and the risk of type 2 diabetes: A two-sample Mendelian Randomization analysis in the European population

**DOI:** 10.3389/fendo.2022.964132

**Published:** 2022-11-03

**Authors:** Ming-Gang Deng, Han-Tao Cui, Yong-Bing Lan, Jia-Qi Nie, Yue-Hui Liang, Chen Chai

**Affiliations:** ^1^ School of Public Health, Wuhan University, Wuhan, China; ^2^ Department of Epidemiology & Biostatistics, School of Public Health, Peking University, Beijing, China; ^3^ Emergency Center, Hubei Clinical Research Center for Emergency and Resuscitation, Zhongnan Hospital of Wuhan University, Wuhan, China

**Keywords:** physical activity, sedentary behaviors, type 2 diabetes, Mendelian Randomization, causal effect

## Abstract

Physical activity (PA) and sedentary behaviors (SB) have been linked to the risk of type 2 diabetes (T2DM) in observational studies; however, it is unclear whether these associations are causative or confounded. This study intends to use summary genetic data from the UK Biobank and other consortiums in conjunction with the two-sample Mendelian Randomization (MR) approach to solve this problem. The inverse variance weighted (IVW) technique was utilized as the primary analysis, with sensitivity analyses using the MR-Egger, weighted-median, and MR-Pleiotropy RESidual Sum and Outlier (PRESSO) techniques. Inverse associations between self-reported moderate PA (OR: 0.3096, 95% CI: 0.1782-0.5380) and vigorous PA (OR: 0.2747, 95% CI: 0.1390-0.5428) with T2DM risk were found, respectively. However, accelerometer-based PA measurement (average acceleration) was not associated with T2DM risk (OR: 1.0284, 95% CI: 0.9831-1.0758). The time (hours/day) spent watching TV was associated with T2DM risk (OR: 2.3490, 95% CI: 1.9084-2.8915), while the time (hours/day) spent using the computer (OR: 0.8496, 95% CI: 0.7178-1.0056), and driving (OR: 3.0679, 95% CI: 0.8448-11.1415) were not associated with T2DM risk. The sensitivity analysis revealed relationships of a similar magnitude. Our study revealed that more PA and less TV viewing were related to a decreased T2DM risk, and provided genetic support for a causal relationship between PA, TV viewing, and T2DM risk.

## Introduction

Type 2 diabetes mellitus (T2DM) is a common disease with increasing incidence globally. The incidence of T2DM in the European region has been continuously rising, from 190/100,000 in 1990 to 328/100,000 in 2019, according to the Global Burden of Disease-2019 (1). Prevention strategies targeting T2DM risk factors are essential to control the growing burden. Among various risk factors for T2DM, including ethnicity, obesity, an unhealthy diet, physical inactivity (PA), sedentary behaviors (SB), and so on (2), the effects of PA and SB have attracted special attention.

PA can provide an antidiabetic impact by improving blood glucose levels in people with T2DM (3), because it entails bodily movements and energy expenditure (4). Numerous prior observational studies have shown both moderate physical activity (MPA) (5, 6) and vigorous physical activity (VPA) (5, 6) were related to decreased risks of T2DM. Additionally, the incidence of T2DM decreased as the quantity of PA increased (7, 8). However, the link between PA and T2DM may not always be plausible, and it may be influenced by gender and the kind of PA. According to a study in Korean adults, working PA was not associated with T2DM risk, whereas transport PA was solely linked to T2DM in males (9).

SB is frequently characterized as sitting, watching television, or couch time, and is a potentially significant factor in health (10). SB was claimed to affect T2DM risk in many studies (11–15). However, no connection between the incidence of T2DM and objectively measured sedentary time was found by an earlier study (16). A population-based study found that watching TV was the main SB, accounting for roughly half of the total sedentary time, followed by computer viewing (17). The majority of studies used overall sedentary time or time spent watching television as the main indicator of SB (13, 15). Nevertheless, mentally active SB, like using a computer or driving (18), may have different impacts on T2DM risk than mentally passive SB, like watching TV, and to our knowledge, this issue has not yet received much attention.

Mendelian randomization (MR), is a well-established tool for causal inference by employing genetic variants as instrumental variables (IV) for exposures, e.g., PA and SB (19). Since the genetic variants are randomly assigned during meiosis, they are not susceptible to reverse causation bias and confounders, which are the general flaws of conventional epidemiological methods (20). Therefore, MR can yield stronger evidence by assessing whether the observed connection between risk variables and outcome is compatible with causal impact.

The objective of this study is to employ the MR technique to pinpoint the causal relationships among PA, SB, and T2DM in the European population. We hypothesis that more physical activity and less sedentary time were related to a lower risk of T2DM.

## Methods

### Study design

**Figure 1:**
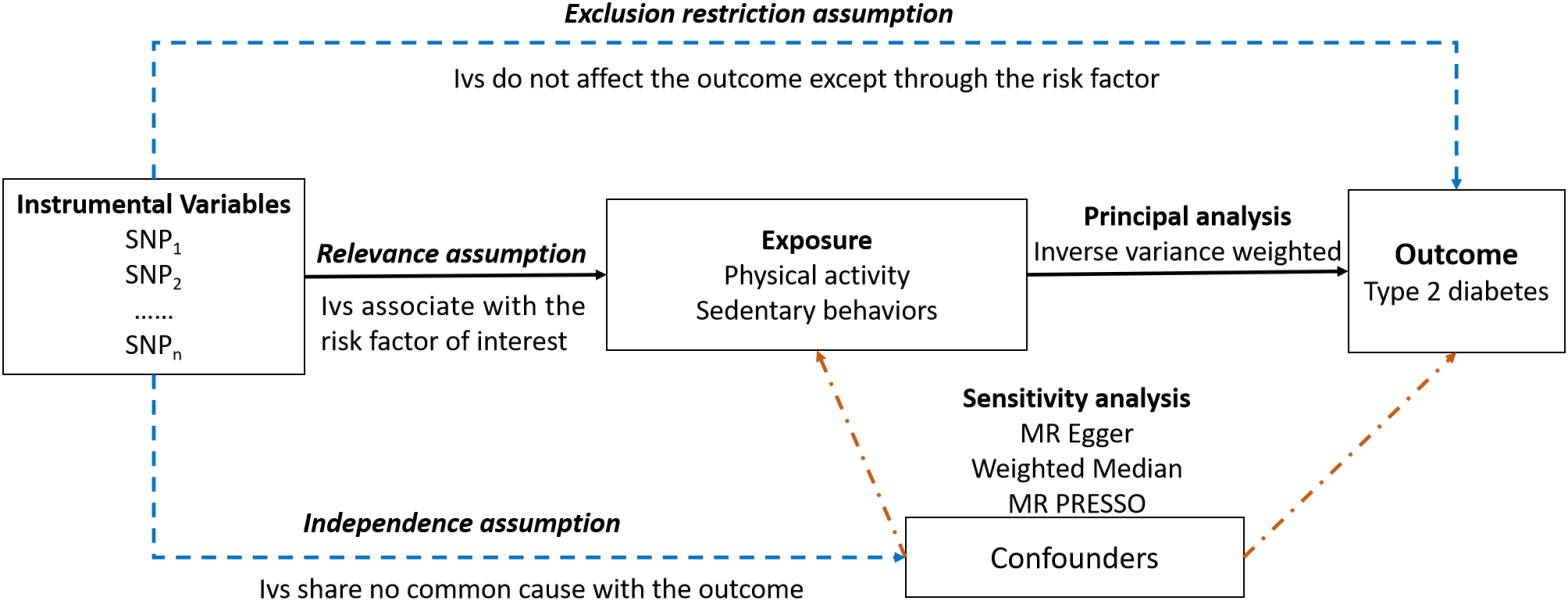
The overview of the study design.

An overview of the study design is presented in **Figure 1**. Our study consisted of five components (1): identification of genetic variants to serve as instrumental variables for PA and SB (2). obtaining the instrumental SNPs for outcome summary data from genome-wide association studies (GWAS) of T2DM (3). harmonizing the exposure and outcome databases (4). performing MR analysis (5) evaluation of MR analysis assumptions and sensitivity analysis.

Three critical assumptions must be met for causal estimations derived from MR analysis to be valid (1): they associate with the risk factor of interest (the relevance assumption) (2); they share no common cause with the outcome (the independence assumption) (3); they do not affect the outcome except through the risk factor (the exclusion restriction assumption) (21).

### GWAS summary data for outcome

Summary data on the associations of genetic variants with physician diagnosed T2DM were obtained from a recent GWAS meta-analysis of 62,892 T2DM patients and 596,424 controls of European ancestry with a total of 16 million gene variations (22). The study was composed of three contributing studies, including the full cohort release of the UK Biobank (UKB), Genetic Epidemiology Research on Aging (GERA), and Diabetes Genetics Replication and Meta-analysis (DIAGRAM).

### GWAS summary data for exposures

#### Physical activities

Three types of PA were included in our study, e.g., self-reported moderate physical activity (MPA), self-reported vigorous physical activity (VPA), and accelerometer-based physical activity (Accelerometer-based PA). Self-reported levels of PA were measured *via* a touchscreen questionnaire, in a fashion similar to the International Physical Activity Questionnaire (23). For MPA, participants were directly asked “How many minutes did you usually spend doing moderate activities on a typical day, like carrying light loads, cycling at normal pace (Do not include walking)?”. A total of 343,827 participants were included.

For VPA, participants were asked “In a typical WEEK, how many days did you do 10 minutes or more of vigorous physical activity? (These are activities that make you sweat or breathe hard such as fast cycling, aerobics, heavy lifting)”, and those who indicated 1 or more such days were then asked “How many minutes did you usually spend doing vigorous activities on a typical DAY”. These participants were then dichotomized into two groups (1): those who reported 0 days of VPA, and (2) those who reported both 3 or more days of VPA and a typical duration of VPA that is 25 minutes or greater. Individuals that did not fall into either of these two groups were excluded, and a total of 261,055 individuals were included, with 98,060 cases and 162,995 controls (24).

Accelerometer-based PA was assessed by an Axivity AX3 wrist-worn accelerometer, as previously described (25). Participants were informed in the invitation email and device mail-out letter that the accelerometer should be worn continuously and that they should carry on with their normal activities. PA information (overall acceleration average) was extracted from 100Hz raw triaxial acceleration data after calibration, removal of gravity and sensor noise, and identification of wear/non-wear episodes. Individuals with less than 3 days (72 hours) of data, or those not having data in each one hour of the 24-hour cycle, and outliers with values more than 4 standard deviations above the mean were excluded. 72 hours of wear was determined to be needed to be within 10% of a complete severe-day measure, which was based on missing data simulations by Doherty et al (25). A total of 91,084 participants were included, with the mean and standard deviation of average acceleration being 27.98 and 8.14, respectively.

#### Sedentary behaviors

Watching television, using the computer, and driving were identified as three types of SB in our study. For the ascertainment of sedentary time, during the first visit, participants were asked three questions, “On a typical day, how many hours do you spend watching TV?”, “In a typical day, how many hours do you spend using the computer? (Do not include using a computer at work)” and “On a typical day, how many hours do you spend driving?”. A total number of 437,887, 360,895, and 310,555 individuals were included for watching TV, using the computer, and driving, respectively. The hours/day of these sedentary behaviors were treated as exposure measurements.

### Selection of instrumental variables

The genome-wide significance level was defined at *p* < 5*10^-8^ to fulfill the relevance assumption, making instrumental variables robustly associated with the outcome. The *F*-statistic was calculated to estimate the strength of each SNP, utilizing the formula given previously (26). To fulfill the independence assumption, we employed the PLINK clumping method (27) to clump the SNPs and make them independent of each other (*r* ^2^ < 0.001, region size = 10000kb). Pleiotropy tests were also carried out to ensure that the exclusion restriction assumption was met (21).

SNPs that were unavailable in the outcome datasets were replaced by suitable proxy SNPs with minimum linkage disequilibrium *R* ^2^ = 0.8 and minor allele frequency threshold = 0.3, where available. Finally, 5, 7, 6, 89, 73, and 6 SNPs associated with MPA, VPA, Accelerometer-based PA, watching television, using the computer, and driving were identified, respectively. Summary statistics are presented in **Supplementary Table S1**.

### Statistical analysis

The SNP-exposure and SNP-outcome coefficients were combined in a random-effects meta-analysis using the inverse-variance weighted (IVW) method to primarily provide an overall estimate of a causal impact.

Several sensitivity analyses were carried out to check and correct the presence of pleiotropy in the causal estimates. Cochran’s *Q* was calculated to check the heterogeneity of the individual causal effect, with a *P*-value < 0.05, indicating the presence of pleiotropy. Consequently, weights were penalized to improve the robustness of the IVW method (28). MR-Egger intercept term was used to assess the horizontal pleiotropy, where deviation from zero indicates the directional pleiotropy. Moreover, the slope of the MR-Egger regression gives valid MR estimates when horizontal pleiotropy exists (29, 30). The complementary weighted-median method was used which can give valid MR estimates by assuming that at least 50% of IVs are effective and ordering the MR estimates of each IV weighted for the inverse of their variance (31). MR pleiotropy residual sum and outlier (MR-PRESSO) global test method was conducted to detect horizontal pleiotropy and the MR-PRESSO outlier test was conducted to correct the horizontal pleiotropy *via* outlier removal (32). The leave-one-out analysis was performed to assess the influence of a single SNP on the MR estimates.

The TwoSampleMR (version 0.5.6), MendelianRandomization (version 0.5.1), and MRPRESSO (version 1.0) packages were used for statistical analyses in R software (version 4.1.0, R Foundation for Statistical Computing). All statistical tests were two-tailed, and significance was considered at a Bonferroni corrected *p-value* below 0.0083 (correcting for 6 exposures and 1 outcome).

## Results

### MR estimates of causal effects of physical activity on type 2 diabetes

MR estimates between PA and T2DM risk are illustrated in **Table 1**. Increment in genetically predicted duration of MPA was associated with lower risk of T2DM (odds ratio [OR]: 0.3096, 95% confidence interval [95% CI]: 0.1782-0.5380, *P*-value < 0.0001). An inverse relationship was also found between VPA and T2DM risk, compared to those who reported 0 days of VPA per week, people who reported both 3 or more days of VPA per week had about 63% lower risk of T2DM (OR: 0.2747, 95% CI: 0.1390-0.5428, *P*-value = 0.0002). However, no significant association between Accelerometer-based PA and T2DM risk was found (OR: 1.0284, 95% CI: 0.9831-1.0758, *P*-value = 0.2290). There was some evidence of heterogeneity based on Cochran’s *Q* (*Q*-value = 22.4315, *P*-value = 0.0004) for the accelerometer-based PA analysis. Consequently, weights were penalized for the IVW method.

**Table 1:** MR estimates between physical activity and T2DM risk.

Methods	OR	95% CI	*P*-value	*Q*-value	*P*-value for heterogeneity ^‡^ or pleiotropy^§^
**MPA**					
IVW	0.3096	0.1782-0.5380	<0.0001	1.9273	0.7491
MR-Egger	1.4053	0.0720-27.4441	0.8369		0.3847
Weighted-Median	0.3167	0.1529-0.6560	0.0019		
MR-PRESSO	0.3096	0.2110-0.4544	0.0039		
**VPA**					
IVW	0.2747	0.1390-0.5428	0.0002	3.1608	0.7884
MR-Egger	0.3445	0.0016-74.8385	0.7138		0.9370
Weighted-Median	0.2569	0.1070-0.6168	0.0024		
MR-PRESSO	0.2747	0.1676-0.4503	0.0021		
**Accelerometer-based PA**					
IVW ^†^	1.0284	0.9831-1.0758	0.2290	22.4315	0.0004
MR-Egger	1.1969	0.8054-1.7786	0.4241		0.4222
Weighted-Median	1.0228	0.9803-1.0671	0.2981		
MR-PRESSO	1.0015	0.9414-1.0654	0.9632		

MPA: moderate physical activity; VPA: vigorous physical activity; OR: odds ratio; CI: confidence intervals; Q-value: Cochran’s Q statistic; IVW: inverse variance weighted

^†^ Weights were penalized due to the presence of heterogeneity based on Cochran’s Q statistic

^‡^ P-value for heterogeneity based on Cochran’s Q statistic

^§^ P-value or pleiotropy based on MR-Egger interceptTable 2 MR estimates between sedentary behavior and T2DM risk

**Figure 2:**
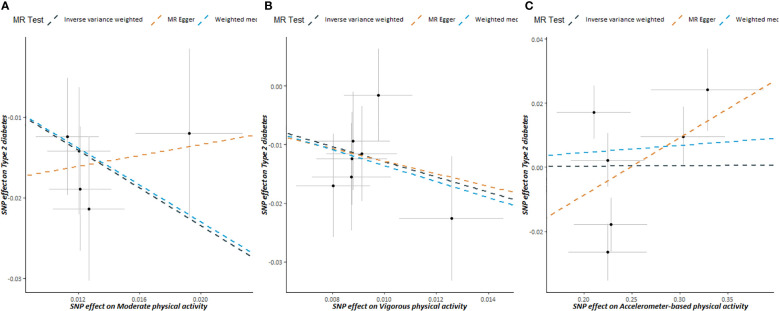
Scatter plots showing the correlation of genetic associations of physical activity with genetic associations with type 2 diabetes, **(A)** moderate physical activity; **(B)** vigorous physical activity; **(C)** accelerometer-based physical activity.

The scatter plots of PA and T2DM risk are depicted in **Figure 2**. MR estimates for each SNP associated with PA in relation to T2DM risk are presented in **Supplementary Figure S1**, and the funnel plots of PA and T2DM risk association are presented in **Supplementary Figure S2**.

### MR estimates of causal effects of sedentary behaviors on type 2 diabetes

MR estimates between SB and T2DM risk are illustrated in **Table 2**. A positive association between the duration of watching TV and T2DM risk was found (OR: 2.3490, 95% CI: 1.9084-2.8915, *P*-value < 0.0001). However, we found no association between the duration of using the computer or driving and T2DM risk. There was some evidence of heterogeneity based on Cochran’s Q for the three types of SB; consequently, for these IVW models, weights were penalized to improve the robustness.

**Table 2:** MR estimates between sedentary behavior and T2DM risk.

Methods	OR	95% CI	*P*-value	*Q*-value	*P*-value for heterogeneity ^‡^ or pleiotropy ^§^
**Watching TV**					
IVW ^†^	2.3490	1.9084-2.8915	<0.0001	283.8163	<0.0001
MR-Egger	4.5997	1.0169-20.8051	0.0470		0.4226
Weighted-Median	2.5143	1.9260-3.2824	<0.0001		
MR-PRESSO ^¶^	2.6092	2.0432-3.3320	<0.0001		
**Using the computer**					
IVW ^†^	0.8496	0.7178-1.0056	0.0580	111.9547	0.0018
MR-Egger	1.6372	0.5794-4.6265	0.3520		0.3083
Weighted-Median	0.8834	0.6982-1.1176	0.3030		
MR-PRESSO ^¶^	0.9120	0.7555-1.1010	0.3411		
**Driving**					
IVW ^†^	3.0679	0.8448-11.1415	0.0880	35.14185	<0.0001
MR-Egger	0.0533	0.0000-73665.88	0.7052		0.5988
Weighted-Median	3.4418	1.5140-7.8243	0.0030		
MR-PRESSO ^¶^	2.9869	0.6258-14.2560	0.2636		

OR, odds ratio; CI, confidence intervals; Q-value, Cochran’s Q statistic; MR, Mendelian randomization; IVW, inverse variance weighted

^†^ Weights were penalized due to the presence of heterogeneity based on Cochran’s Q statistic

^‡^ P-value for heterogeneity based on Cochran’s Q statistic

^§^ P-value or pleiotropy based on MR-Egger intercept

^¶^ Results after removal of outliers

**Figure 3:**
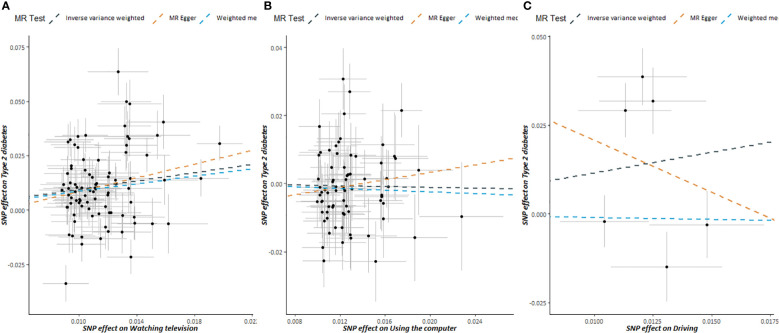
Scatter plots showing the correlation of genetic associations of sedentary behavior with genetic associations with type 2 diabetes, **(A)** watching television; **(B)** using the computer; **(C)** driving.

Scatter plots of SB and T2DM risk are presented in **Figure 3**. MR estimates for each SNP associated with SB in relation to T2DM risk are presented in **Supplementary Figure S3**, and the funnel plots of SB and T2DM risk association are presented in **Supplementary Figure S4**.

### Evaluation of assumptions and sensitivity analyses

The strength of the genetic instruments denoted by the *F*-statistic was ≥ 10 for all the exposures, see **Supplementary Table S1**. No evidence of directional pleiotropy was found for all exposures (all MR-Egger intercept *P*-values > 0.05), see **Tables 1** and **2**. The estimates from the weighted-median method for the exposures were consistent with those of IVW methods, with an exception for driving (**Tables 1**, **2**). The MR-PRESSO approach identified outlier SNPs for watching TV, using the computer, and driving, but similar magnitude associations were found after these outliers were excluded from the analysis (**Table 1**, **2**). Moreover, the estimates from the leave-one-out analysis did not reveal any influential SNPs driving the overall association (**Supplementary Figures S5**, **S6**).

## Discussion

The present study performed a Mendelian Randomization in the European population to investigate the genetic association of physical activity and sedentary behaviors with the risk of type 2 diabetes. Our study validated that self-reported physical activity, either moderate or vigorous intensity, is genetically connected with lower risks of developing T2DM, whereas acceleration-based physical activity is not. Watching TV is correlated with an elevated risk of developing T2DM, while using a computer, and driving are not.

Our findings that MPA and VPA are associated with a lower risk of T2DM are in line with those of previous studies. According to a previous study, those who regularly engage in MPA had a 30% lower risk of T2DM compared to sedentary individuals (33).. Weekly VPA may decrease the risk of T2DM among those who are normal weight or overweight (34).. However, the link between PA and T2DM may not always be plausible, and it may be influenced by gender and the kind of PA. According to a study in Korean adults, wsorking PA was not associated with T2DM risk, whereas transport PA was solely linked to T2DM in males (9). One previous MR study reported that objectively measured average or vigorous physical activity and SB are not associated with the risk of T2DM (16). This outcome is in line with our findings that there is no correlation between acceleration-based PA and T2DM risk. Acceleration-based PA and self-reported PA were demonstrated to have a poor agreement in several studies (35, 36). In general adults, the self-reported time spent on MPA and VPA exceeded the time measured with the accelerometer (36). The disparity between self-reported and Accelerometer-measured MVPA increased with higher activity and intensity levels (35). The PA acquired from the accelerometer and the self-report is not conceptually equivalent, which explains why the Acceleration-based PA findings in our research vary from the MPA and VPA (37).

Numerous observational studies reported that watching TV is associated with a higher incidence of T2DM (38–40), but the finding was subject to unobserved confounders, or reverse causation. For the first time, the present study confirms this association *via* Mendelian Randomization, which avoids residual confounding by taking advantage of the instrument variable of genetic variants. A total of 89 genetic SNPs were identified to be associated with watching TV, which enables a robust estimation of the association between watching TV and the risk of T2DM. On average, watching TV increases T2DM more than one-fold.

The present study did not find computer usage was associated with the risk of T2DM. The finding concurs with previous studies. One study showed that computer use was unrelated to HbA1c and blood lipids (41). Computer use also had no significant association with the risk of T2DM in Taiwanese older adults (42). It differs from watching television in that individuals may be more physically and cognitively engaged while using a computer than when watching television (43). Another possible explanation is that the computer time used in this article does not include computer time at work, which may be biased.

The present study did not find driving was associated with the risk of T2DM. Most recent research on driving and T2DM, to our knowledge, have focused on occupational drivers (44–46), who had a higher prevalence of T2DM. However, the average daily driving duration of participants in our study was roughly 1.18 hours, indicating that the driving employment may unlikely impact our findings. Furthermore, it is commonly understood that driving necessitates drivers to be cognitively engaged, which is not the case while watching television (18).

Several mechanisms could explain the links. First, the disparate impacts of TV viewing and physical activity may be mediated by diet and BMI. PA can reduce the risk of T2DM by reducing obesity, which is a greater risk of T2DM (47, 48). Individuals who engage in more vigorous physical activity may adhere to a healthier diet more closely, consume fewer snacks, and spend less time watching television (49). Second, according to a prospective study, PA played an important role in glycemic control (50), and the skeletal muscle, which is a primary tissue that determines blood glucose, by increasing insulin sensitivity (51, 52). Nevertheless, SB is not conducive to glycemic control (53). Additionally, PA’s antioxidant and anti-inflammatory properties may help suppress the progress of T2DM (54).

There are certain strengths of our study. We apply the MR method to avoid confounding biases and reverse causation in observational studies. The genetic link between PA, SB, and T2DM risk was validated by our study. To our knowledge, we are the first to explore the genetic relationship between SB and T2DM from the angles of watching TV, using a computer, and driving.

Several limitations must be addressed. To begin, our research included only European individuals, which means that the findings cannot be properly extrapolated to other ethnic groups. Moreover, self-reported PA and SB may introduce measurement bias and result in estimations that differ from objective measures. Furthermore, since our research was limited to summary data, populations were not categorized according to sociodemographic factors (e.g., age, sex, or employment) when examining casual connections. Additionally, but certainly not least, light physical activity and other sedentary activities were not considered in the study. Finally, this study was a univariate MR study, without multi-adjustments for BMI, obesity, and other covariates, which were confounders for T2DM risks.

## Conclusion

Our study revealed that more PA and less TV viewing were related to a decreased T2DM risk, and provided genetic support for a causal relationship between PA, TV viewing, and T2DM risk.

## Data availability statement

The data that support the finding of this work was available in the UK Biobank at https://www.ukbiobank.ac.uk/ and the recently published article at https://www.ncbi.nlm.nih.gov/pmc/articles/PMC6195860/. These data were derived from the IEU OpenGWAS Project at https://gwas.mrcieu.ac.uk/, last accessed on 21 February 2022.

## Author contributions

All authors listed have made a substantial, direct, and intellectual contribution to the work and approved it for publication.

## Conflict of interest

The authors declare that the research was conducted in the absence of any commercial or financial relationships that could be construed as a potential conflict of interest.

## Publisher’s note

All claims expressed in this article are solely those of the authors and do not necessarily represent those of their affiliated organizations, or those of the publisher, the editors and the reviewers. Any product that may be evaluated in this article, or claim that may be made by its manufacturer, is not guaranteed or endorsed by the publisher.
