# Supplementary material for: Physical activity, sedentary behavior, and the risk of type 2 diabetes: A two-sample Mendelian Randomization analysis in the European population
Source: Front Endocrinol (Lausanne). 2022 Nov 3;13:964132. doi: 10.3389/fendo.2022.964132 (PMC9670309; doi:10.3389/fendo.2022.964132)
Supplement: Supplementary file 1 [file DataSheet_1.pdf]

Supplementary Material

Supplementary Table S1 Characteristics of the SNPs used in our study

Supplementary Figure S1 Mendelian Randomization analysis for individual SNPs associated with physical activity in relation to type 2 diabetes risk

Supplementary Figure S2 Funnel plots of risk estimates of physical activity and type 2 diabetes

Supplementary Figure S3 Mendelian Randomization analysis for individual SNPs associated with sedentary behavior in relation to type 2 diabetes risk

Supplementary Figure S4 Funnel plots of risk estimates of sedentary behavior and type 2 diabetes

Supplementary Figure S5 Mendelian randomization estimates between physical activity and type 2 diabetes risk by leaving one SNP out at a time

Supplementary Figure S6 Mendelian randomization estimates between sedentary behaviors and type 2 diabetes risk by leaving one SNP out at a time

Supplementary Table S1 Characteristics of the SNPs used in our study

SNP	chr	pos	EA	OA	EAF	N	R^2	F	exposure	exposure			outcome		
										beta	se	pval	beta	se	pval
rs10189857	2	60713235	G	A	0.432	437887	1.91E-04	83.718	Watching television	0.015	0.002	5.70E-20	0.025	0.008	1.17E-03
rs10269099	7	126371011	T	G	0.391	437887	7.17E-05	31.412	Watching television	0.009	0.002	2.10E-08	0.001	0.008	8.63E-01
rs10739499	9	120514261	G	C	0.663	437887	6.86E-05	30.049	Watching television	-0.009	0.002	4.20E-08	-0.010	0.008	2.16E-01
rs10765777	11	95656385	C	A	0.392	437887	1.09E-04	47.730	Watching television	-0.011	0.002	4.90E-12	-0.023	0.008	4.39E-03
rs111901094	19	19513570	T	G	0.182	437887	8.39E-05	36.720	Watching television	0.013	0.002	1.40E-09	0.064	0.011	3.68E-09
rs11191129	10	103606543	T	C	0.423	437887	8.56E-05	37.466	Watching television	-0.010	0.002	9.30E-10	-0.010	0.008	1.89E-01
rs11222919	11	131969663	G	T	0.175	437887	7.71E-05	33.750	Watching television	-0.012	0.002	6.30E-09	0.001	0.010	9.19E-01
rs11245482	10	126733546	C	T	0.385	437887	8.85E-05	38.772	Watching television	0.010	0.002	4.80E-10	0.010	0.008	2.01E-01
rs114755463	5	152503110	A	G	0.168	437887	8.70E-05	38.096	Watching television	0.013	0.002	6.70E-10	0.030	0.011	4.85E-03
rs11662211	18	77618869	T	C	0.506	437887	8.05E-05	35.242	Watching television	-0.009	0.002	2.90E-09	-0.019	0.008	1.37E-02
rs11680095	2	181825956	T	C	0.593	437887	7.22E-05	31.619	Watching television	-0.009	0.002	1.90E-08	-0.001	0.008	8.76E-01
rs11696187	20	58891882	T	C	0.160	437887	8.91E-05	39.029	Watching television	-0.014	0.002	4.20E-10	-0.004	0.011	7.31E-01
rs11700249	20	11910800	G	T	0.409	437887	7.73E-05	33.860	Watching television	0.009	0.002	5.90E-09	0.006	0.008	4.80E-01
rs11714337	3	71582521	A	G	0.430	437887	8.64E-05	37.855	Watching television	-0.010	0.002	7.60E-10	-0.034	0.008	2.12E-05
rs11877758	18	35138110	G	T	0.313	437887	9.43E-05	41.285	Watching television	0.011	0.002	1.30E-10	0.010	0.009	2.28E-01
rs11911112	21	40528346	C	A	0.366	437887	9.65E-05	42.247	Watching television	-0.011	0.002	8.00E-11	-0.009	0.008	2.61E-01
rs12045585	1	243673099	A	G	0.131	437887	8.92E-05	39.068	Watching television	-0.015	0.002	4.10E-10	0.006	0.012	5.98E-01
rs12553324	9	23347865	G	C	0.415	437887	1.61E-04	70.523	Watching television	-0.014	0.002	4.50E-17	0.022	0.008	6.63E-03
rs1291871	10	11086083	C	T	0.514	437887	7.50E-05	32.862	Watching television	0.009	0.002	9.90E-09	0.017	0.008	3.17E-02
rs13014947	2	193742999	A	G	0.575	437887	9.74E-05	42.663	Watching television	0.011	0.002	6.50E-11	0.010	0.008	2.17E-01
rs1324491	1	60350616	A	G	0.131	437887	7.39E-05	32.370	Watching television	0.013	0.002	1.30E-08	0.010	0.012	3.98E-01
rs1727332	12	123718301	T	C	0.754	437887	1.16E-04	50.601	Watching television	0.013	0.002	1.10E-12	0.039	0.009	2.43E-05
rs17789218	6	100600097	C	T	0.244	437887	7.96E-05	34.845	Watching television	-0.011	0.002	3.60E-09	0.003	0.008	7.43E-01
rs178203	14	26959322	C	T	0.750	437887	9.78E-05	42.845	Watching television	0.012	0.002	5.90E-11	0.008	0.009	3.66E-01
rs180396	13	60437497	T	C	0.747	437887	6.85E-05	30.007	Watching television	0.010	0.002	4.30E-08	0.004	0.009	6.34E-01
rs1889996	13	54269950	G	T	0.739	437887	9.97E-05	43.664	Watching television	0.012	0.002	3.90E-11	0.007	0.009	4.35E-01
rs1993092	6	98689604	C	T	0.390	437887	7.78E-05	34.051	Watching television	-0.010	0.002	5.40E-09	0.012	0.008	1.41E-01
rs2073869	9	135763816	T	C	0.167	437887	9.50E-05	41.610	Watching television	-0.014	0.002	1.10E-10	0.003	0.011	7.71E-01
rs2106164	7	92661753	C	T	0.532	437887	7.88E-05	34.513	Watching television	-0.009	0.002	4.20E-09	-0.003	0.008	6.96E-01

rs2185490	14	69732119	C	A	0.618	437887	7.41E-05	32.471	Watching television	0.009	0.002	1.20E-08	0.013	0.008	1.22E-01
rs2240857	7	8010634	G	T	0.141	437887	1.09E-04	47.890	Watching television	0.016	0.002	4.50E-12	0.014	0.011	2.22E-01
rs2283	5	106773623	G	A	0.339	437887	6.99E-05	30.592	Watching television	-0.009	0.002	3.20E-08	-0.007	0.008	3.67E-01
rs2352984	3	49948728	C	T	0.431	437887	3.46E-04	151.702	Watching television	0.020	0.002	7.40E-35	0.031	0.008	1.34E-04
rs249960	5	96164771	G	A	0.182	437887	7.45E-05	32.633	Watching television	-0.012	0.002	1.10E-08	0.008	0.010	4.39E-01
rs262890	5	62930015	G	A	0.299	437887	1.33E-04	58.042	Watching television	0.013	0.002	2.60E-14	0.027	0.009	1.84E-03
rs263771	2	185921692	A	C	0.233	437887	9.27E-05	40.602	Watching television	0.012	0.002	1.90E-10	0.015	0.009	9.75E-02
rs2646351	4	55701312	A	G	0.453	437887	7.10E-05	31.101	Watching television	0.009	0.002	2.40E-08	0.012	0.008	1.26E-01
rs2678662	2	104446759	G	T	0.608	437887	1.06E-04	46.621	Watching television	0.011	0.002	8.60E-12	0.012	0.008	1.46E-01
rs2725371	8	30854033	G	A	0.696	437887	1.34E-04	58.479	Watching television	-0.013	0.002	2.10E-14	-0.050	0.009	4.18E-09
rs2906604	2	107624244	C	T	0.497	437887	9.82E-05	42.994	Watching television	0.010	0.002	5.50E-11	0.018	0.008	1.94E-02
rs3138499	9	92219921	C	A	0.518	437887	1.07E-04	46.658	Watching television	0.011	0.002	8.50E-12	0.009	0.008	2.76E-01
rs34094119	8	10935898	G	A	0.525	437887	7.39E-05	32.382	Watching television	0.009	0.002	1.30E-08	-0.034	0.008	6.23E-05
rs35797019	3	93987306	G	A	0.392	437887	7.32E-05	32.040	Watching television	-0.009	0.002	1.50E-08	-0.031	0.008	9.30E-05
rs3754970	2	162091836	C	T	0.503	437887	8.41E-05	36.828	Watching television	0.010	0.002	1.30E-09	0.008	0.008	2.89E-01
rs3810496	20	62406886	C	T	0.616	437887	7.42E-05	32.482	Watching television	0.009	0.002	1.20E-08	0.032	0.008	4.85E-05
rs4110177	5	88793281	A	G	0.367	437887	7.39E-05	32.375	Watching television	0.009	0.002	1.30E-08	0.020	0.008	1.27E-02
rs4303732	2	100830040	C	T	0.402	437887	1.00E-04	43.779	Watching television	-0.011	0.002	3.70E-11	-0.016	0.008	4.11E-02
rs4339469	6	98369230	G	T	0.629	437887	1.32E-04	57.861	Watching television	0.013	0.002	2.80E-14	0.012	0.008	1.27E-01
rs4469687	1	184679019	G	A	0.484	437887	6.99E-05	30.621	Watching television	0.009	0.002	3.10E-08	0.009	0.008	2.35E-01
rs4567133	9	22606560	A	C	0.810	437887	9.32E-05	40.821	Watching television	-0.013	0.002	1.70E-10	0.003	0.010	8.10E-01
rs4747438	10	22124263	T	C	0.677	437887	1.10E-04	48.152	Watching television	-0.012	0.002	3.90E-12	-0.002	0.009	8.38E-01
rs4788616	16	72211984	G	T	0.391	437887	8.90E-05	38.982	Watching television	-0.010	0.002	4.30E-10	0.012	0.008	1.28E-01
rs4847408	1	93791437	C	G	0.649	437887	8.92E-05	39.068	Watching television	-0.010	0.002	4.10E-10	-0.007	0.008	4.14E-01
rs494566	9	1785717	T	C	0.332	437887	8.20E-05	35.930	Watching television	0.010	0.002	2.00E-09	-0.016	0.008	6.08E-02
rs57555420	1	97783448	T	C	0.282	437887	7.53E-05	32.957	Watching television	0.010	0.002	9.40E-09	0.002	0.009	8.18E-01
rs6102912	20	41202935	C	T	0.410	437887	1.04E-04	45.603	Watching television	-0.011	0.002	1.40E-11	-0.015	0.008	5.50E-02
rs6125907	20	48730315	A	C	0.092	437887	7.85E-05	34.381	Watching television	0.016	0.003	4.50E-09	-0.006	0.014	6.45E-01
rs61864793	10	85803372	C	T	0.252	437887	8.42E-05	36.894	Watching television	-0.011	0.002	1.20E-09	-0.010	0.009	2.87E-01
rs62145951	2	68399586	C	T	0.263	437887	1.01E-04	44.433	Watching television	-0.012	0.002	2.60E-11	0.010	0.009	2.54E-01
rs62199883	2	215376706	A	C	0.486	437887	1.73E-04	75.768	Watching television	0.014	0.002	3.20E-18	-0.006	0.008	4.39E-01
rs6493583	15	53096084	G	C	0.138	437887	7.52E-05	32.936	Watching television	0.013	0.002	9.50E-09	0.034	0.011	3.03E-03
rs6511708	19	10788813	C	T	0.665	437887	1.17E-04	51.410	Watching television	-0.012	0.002	7.50E-13	0.001	0.008	8.96E-01

rs68056254	2	147846855	T	G	0.151	437887	8.40E-05	36.792	Watching television	0.013	0.002	1.30E-09	0.049	0.011	7.25E-06
rs6814554	4	152454334	A	G	0.474	437887	1.64E-04	71.822	Watching television	0.014	0.002	2.40E-17	0.015	0.008	6.20E-02
rs6850494	4	82291771	C	A	0.386	437887	7.98E-05	34.938	Watching television	0.010	0.002	3.40E-09	0.030	0.008	1.75E-04
rs6895658	5	124274035	C	T	0.193	437887	9.50E-05	41.583	Watching television	-0.013	0.002	1.10E-10	0.010	0.010	3.14E-01
rs6994132	8	92653740	C	T	0.579	437887	8.73E-05	38.250	Watching television	-0.010	0.002	6.20E-10	-0.003	0.008	6.82E-01
rs7089973	10	116569565	A	C	0.380	437887	7.28E-05	31.892	Watching television	0.009	0.002	1.60E-08	-0.011	0.008	1.61E-01
rs7184800	16	53509131	A	G	0.303	437887	1.38E-04	60.476	Watching television	-0.013	0.002	7.40E-15	-0.033	0.009	1.09E-04
rs72673939	8	118867693	C	G	0.183	437887	7.89E-05	34.557	Watching television	0.012	0.002	4.10E-09	0.017	0.010	8.79E-02
rs73571431	9	126136139	T	C	0.110	437887	8.79E-05	38.495	Watching television	0.016	0.003	5.50E-10	0.041	0.013	1.21E-03
rs749056	1	110037838	G	T	0.304	437887	7.65E-05	33.481	Watching television	-0.010	0.002	7.20E-09	-0.005	0.008	5.49E-01
rs749671	16	31088347	A	G	0.372	437887	1.04E-04	45.388	Watching television	-0.011	0.002	1.60E-11	-0.005	0.007	4.67E-01
rs75499503	6	26145217	T	C	0.220	437887	2.04E-04	89.463	Watching television	-0.018	0.002	3.10E-21	-0.015	0.009	8.27E-02
rs75641275	1	98327133	C	A	0.143	437887	1.06E-04	46.239	Watching television	0.015	0.002	1.00E-11	0.034	0.011	1.98E-03
rs7708324	5	147920094	G	A	0.377	437887	8.31E-05	36.404	Watching television	-0.010	0.002	1.60E-09	-0.029	0.008	4.05E-04
rs7798292	7	112974602	A	G	0.435	437887	8.40E-05	36.764	Watching television	-0.010	0.002	1.30E-09	-0.012	0.008	1.33E-01
rs7899206	10	127188859	G	T	0.492	437887	8.42E-05	36.852	Watching television	-0.010	0.002	1.30E-09	-0.004	0.008	5.83E-01
rs7921305	10	133775196	A	G	0.253	437887	8.96E-05	39.257	Watching television	-0.011	0.002	3.70E-10	0.013	0.009	1.49E-01
rs801733	11	65934549	C	A	0.358	437887	1.30E-04	56.723	Watching television	-0.012	0.002	5.00E-14	-0.014	0.008	8.28E-02
rs814197	1	61092456	G	T	0.467	437887	1.03E-04	44.948	Watching television	-0.011	0.002	2.00E-11	-0.001	0.008	9.27E-01
rs872169	2	24259188	G	C	0.206	437887	7.59E-05	33.233	Watching television	0.011	0.002	8.20E-09	-0.002	0.010	8.50E-01
rs883027	2	50600165	C	G	0.420	437887	8.22E-05	36.016	Watching television	0.010	0.002	2.00E-09	-0.005	0.008	5.15E-01
rs9300594	13	100869905	G	A	0.254	437887	8.56E-05	37.504	Watching television	0.011	0.002	9.10E-10	0.012	0.009	1.70E-01
rs9471333	6	40362023	T	C	0.552	437887	9.73E-05	42.592	Watching television	-0.010	0.002	6.70E-11	-0.035	0.008	1.25E-05
rs9834970	3	36856030	C	T	0.498	437887	7.19E-05	31.491	Watching television	-0.009	0.002	2.00E-08	-0.012	0.008	1.30E-01
rs9867437	3	85676752	C	A	0.460	437887	9.45E-05	41.366	Watching television	-0.010	0.002	1.30E-10	-0.023	0.008	3.57E-03
rs9880023	3	54178199	T	G	0.556	437887	8.22E-05	35.994	Watching television	0.010	0.002	2.00E-09	-0.002	0.008	7.92E-01
rs996234	5	59455212	A	G	0.516	437887	9.34E-05	40.916	Watching television	-0.011	0.002	1.60E-10	-0.004	0.008	6.40E-01
rs10208088	2	221055873	T	C	0.580	360895	8.50E-05	30.685	Using the computer	-0.010	0.002	3.00E-08	-0.009	0.008	2.49E-01
rs10518019	4	67959875	G	A	0.476	360895	8.24E-05	29.738	Using the computer	0.010	0.002	4.90E-08	-0.005	0.008	5.01E-01
rs10828248	10	21824619	G	A	0.345	360895	8.55E-05	30.846	Using the computer	0.011	0.002	2.80E-08	-0.002	0.008	8.16E-01
rs11259902	15	83886529	A	C	0.200	360895	8.84E-05	31.895	Using the computer	0.013	0.002	1.60E-08	-0.015	0.010	1.24E-01
rs112600282	2	156895797	G	A	0.110	360895	9.28E-05	33.481	Using the computer	-0.017	0.003	7.20E-09	-0.007	0.013	5.60E-01
rs113851275	9	98297220	A	G	0.108	360895	1.11E-04	39.994	Using the computer	0.019	0.003	2.50E-10	-0.016	0.013	2.09E-01

rs11634155	15	26693096	C	T	0.335	360895	9.85E-05	35.556	Using the computer	-0.012	0.002	2.50E-09	-0.011	0.008	1.53E-01
rs117405403	1	197744098	C	G	0.585	360895	1.10E-04	39.557	Using the computer	0.012	0.002	3.20E-10	-0.001	0.008	9.07E-01
rs11749912	5	88065628	G	A	0.575	360895	1.34E-04	48.256	Using the computer	-0.013	0.002	3.70E-12	-0.003	0.008	7.13E-01
rs11766392	7	69838127	T	G	0.285	360895	1.10E-04	39.584	Using the computer	-0.013	0.002	3.10E-10	0.008	0.009	3.46E-01
rs11942953	4	163753973	C	T	0.537	360895	8.96E-05	32.324	Using the computer	-0.010	0.002	1.30E-08	0.019	0.008	1.53E-02
rs12128707	1	72588119	G	A	0.264	360895	9.29E-05	33.517	Using the computer	0.012	0.002	7.10E-09	0.013	0.008	1.05E-01
rs12145677	1	110023610	A	G	0.297	360895	1.68E-04	60.762	Using the computer	0.016	0.002	6.40E-15	-0.005	0.009	5.58E-01
rs12521638	5	166458770	G	A	0.447	360895	8.33E-05	30.056	Using the computer	0.010	0.002	4.20E-08	0.001	0.008	8.56E-01
rs12553324	9	23347865	G	C	0.416	360895	2.47E-04	89.026	Using the computer	0.017	0.002	3.90E-21	0.022	0.008	6.63E-03
rs12706626	7	124531370	A	G	0.384	360895	8.52E-05	30.762	Using the computer	0.010	0.002	2.90E-08	-0.005	0.008	5.17E-01
rs12820967	12	38921745	C	T	0.322	360895	1.01E-04	36.556	Using the computer	0.012	0.002	1.50E-09	0.012	0.009	1.47E-01
rs12946454	17	43208121	T	A	0.268	360895	9.73E-05	35.123	Using the computer	-0.012	0.002	3.10E-09	0.017	0.008	3.42E-02
rs13262595	8	143316970	G	A	0.561	360895	2.00E-04	72.370	Using the computer	0.016	0.002	1.80E-17	0.006	0.008	4.46E-01
rs13422733	2	102010245	T	C	0.126	360895	8.34E-05	30.115	Using the computer	-0.015	0.003	4.10E-08	0.023	0.012	5.34E-02
rs136553	22	27255675	T	C	0.377	360895	9.97E-05	35.994	Using the computer	0.011	0.002	2.00E-09	-0.007	0.008	3.94E-01
rs1395020	4	139690326	A	G	0.303	360895	8.44E-05	30.465	Using the computer	-0.011	0.002	3.40E-08	0.007	0.009	4.29E-01
rs1448355	11	131286685	T	C	0.618	360895	1.17E-04	42.262	Using the computer	0.012	0.002	8.00E-11	-0.009	0.008	2.82E-01
rs1469249	5	113837198	A	G	0.211	360895	9.22E-05	33.277	Using the computer	-0.013	0.002	8.00E-09	0.016	0.010	9.48E-02
rs1648906	18	35311651	A	G	0.321	360895	8.71E-05	31.428	Using the computer	-0.011	0.002	2.10E-08	0.015	0.009	8.37E-02
rs166835	15	47716037	T	C	0.556	360895	9.55E-05	34.481	Using the computer	-0.011	0.002	4.30E-09	0.008	0.008	2.93E-01
rs16912540	11	13271422	G	A	0.137	360895	9.80E-05	35.363	Using the computer	-0.016	0.003	2.70E-09	0.010	0.011	3.65E-01
rs17167210	7	133339343	A	G	0.436	360895	1.06E-04	38.306	Using the computer	-0.011	0.002	6.00E-10	-0.001	0.008	9.03E-01
rs17789218	6	100600097	C	T	0.245	360895	9.94E-05	35.872	Using the computer	0.013	0.002	2.10E-09	0.003	0.008	7.43E-01
rs17862355	7	126970135	G	T	0.442	360895	1.03E-04	37.243	Using the computer	-0.011	0.002	1.00E-09	-0.005	0.008	5.46E-01
rs1987942	13	54004785	C	T	0.617	360895	9.43E-05	34.046	Using the computer	-0.011	0.002	5.40E-09	-0.010	0.008	2.42E-01
rs1999244	13	66821625	T	A	0.220	360895	9.72E-05	35.065	Using the computer	0.013	0.002	3.20E-09	-0.002	0.009	8.67E-01
rs2032780	2	215073935	C	T	0.406	360895	1.20E-04	43.374	Using the computer	0.012	0.002	4.50E-11	0.005	0.008	5.57E-01
rs2041687	2	60405620	G	T	0.549	360895	1.09E-04	39.230	Using the computer	0.012	0.002	3.80E-10	0.009	0.008	2.74E-01
rs2068625	4	159856739	C	T	0.698	360895	1.41E-04	50.776	Using the computer	0.014	0.002	1.00E-12	0.001	0.009	8.80E-01
rs206965	12	120856332	C	T	0.791	360895	9.23E-05	33.296	Using the computer	-0.013	0.002	7.90E-09	-0.008	0.009	3.80E-01
rs2120461	1	8447722	T	C	0.661	360895	1.11E-04	40.002	Using the computer	0.012	0.002	2.50E-10	-0.013	0.008	1.20E-01
rs2220599	5	7378854	G	C	0.369	360895	1.17E-04	42.140	Using the computer	0.012	0.002	8.50E-11	-0.005	0.008	5.54E-01
rs2588543	4	37000406	T	C	0.672	360895	8.67E-05	31.287	Using the computer	0.011	0.002	2.20E-08	-0.003	0.008	7.46E-01

rs2734833	11	113292920	A	G	0.607	360895	1.21E-04	43.725	Using the computer	0.012	0.002	3.80E-11	-0.009	0.008	2.65E-01
rs2748985	1	1853184	C	T	0.544	360895	1.29E-04	46.447	Using the computer	0.012	0.002	9.40E-12	0.001	0.008	8.84E-01
rs2756121	14	103989581	A	T	0.367	360895	1.03E-04	37.002	Using the computer	-0.012	0.002	1.20E-09	0.013	0.008	1.17E-01
rs2761438	1	110752139	G	A	0.624	360895	1.00E-04	36.273	Using the computer	-0.011	0.002	1.70E-09	-0.001	0.008	9.55E-01
rs28710456	4	152667171	C	T	0.490	360895	8.98E-05	32.426	Using the computer	-0.010	0.002	1.20E-08	0.008	0.008	2.84E-01
rs306755	20	3099752	C	T	0.475	360895	8.92E-05	32.178	Using the computer	0.010	0.002	1.40E-08	-0.003	0.008	7.46E-01
rs34238696	5	161356241	G	A	0.107	360895	8.94E-05	32.268	Using the computer	-0.017	0.003	1.30E-08	-0.008	0.013	5.26E-01
rs3730399	16	67229019	G	A	0.064	360895	1.04E-04	37.507	Using the computer	-0.023	0.004	9.10E-10	0.010	0.016	5.42E-01
rs422115	5	102634340	T	A	0.251	360895	9.34E-05	33.698	Using the computer	0.012	0.002	6.40E-09	0.031	0.009	6.34E-04
rs4704043	5	72159179	T	C	0.714	360895	9.16E-05	33.075	Using the computer	0.012	0.002	8.90E-09	-0.002	0.009	8.05E-01
rs4852252	2	71539301	C	T	0.564	360895	8.51E-05	30.718	Using the computer	0.010	0.002	3.00E-08	-0.001	0.008	8.99E-01
rs56229818	15	58662232	C	T	0.484	360895	8.51E-05	30.715	Using the computer	-0.010	0.002	3.00E-08	-0.008	0.008	2.84E-01
rs58638214	19	31864938	T	C	0.398	360895	1.31E-04	47.117	Using the computer	-0.013	0.002	6.70E-12	-0.027	0.008	8.03E-04
rs6028090	20	59856465	A	G	0.555	360895	1.27E-04	45.870	Using the computer	0.013	0.002	1.30E-11	-0.007	0.008	4.08E-01
rs613872	18	53210302	T	G	0.827	360895	1.17E-04	42.329	Using the computer	-0.016	0.002	7.70E-11	0.003	0.010	7.45E-01
rs6449708	5	50851575	C	T	0.532	360895	9.23E-05	33.302	Using the computer	-0.011	0.002	7.90E-09	0.011	0.008	1.69E-01
rs6780848	3	8179920	G	T	0.270	360895	8.46E-05	30.541	Using the computer	0.011	0.002	3.30E-08	-0.006	0.009	4.66E-01
rs6935828	6	140811367	T	C	0.556	360895	8.49E-05	30.650	Using the computer	0.010	0.002	3.10E-08	0.017	0.008	3.35E-02
rs707926	6	31748820	A	G	0.150	360895	9.02E-05	32.543	Using the computer	0.015	0.003	1.20E-08	-0.015	0.011	1.63E-01
rs7209653	17	19882084	C	T	0.295	360895	1.24E-04	44.772	Using the computer	-0.013	0.002	2.20E-11	-0.008	0.009	3.49E-01
rs7281293	21	34291496	C	A	0.248	360895	9.75E-05	35.189	Using the computer	0.013	0.002	3.00E-09	0.002	0.008	7.82E-01
rs72828532	6	19065342	C	T	0.179	360895	1.20E-04	43.367	Using the computer	0.016	0.002	4.50E-11	-0.004	0.010	6.99E-01
rs72847500	6	37643909	C	T	0.121	360895	8.76E-05	31.623	Using the computer	0.016	0.003	1.90E-08	0.012	0.012	3.40E-01
rs7288455	22	39966547	G	A	0.567	360895	9.05E-05	32.647	Using the computer	-0.011	0.002	1.10E-08	0.023	0.008	4.31E-03
rs73578186	9	126334485	T	C	0.324	360895	1.09E-04	39.264	Using the computer	-0.012	0.002	3.70E-10	0.001	0.008	8.71E-01
rs7526112	1	93747683	G	T	0.362	360895	9.05E-05	32.653	Using the computer	-0.011	0.002	1.10E-08	0.010	0.008	2.12E-01
rs7564844	2	215335556	A	G	0.701	360895	1.07E-04	38.453	Using the computer	-0.012	0.002	5.60E-10	-0.021	0.009	1.54E-02
rs76112266	2	201087157	G	C	0.166	360895	1.22E-04	43.862	Using the computer	-0.016	0.002	3.50E-11	0.001	0.011	9.33E-01
rs7630869	3	49522543	T	C	0.304	360895	1.84E-04	66.249	Using the computer	0.016	0.002	4.00E-16	0.002	0.009	8.63E-01
rs7904398	10	67954193	T	C	0.503	360895	8.63E-05	31.135	Using the computer	-0.010	0.002	2.40E-08	0.003	0.008	6.63E-01
rs7968738	12	90281747	A	G	0.264	360895	9.88E-05	35.659	Using the computer	-0.012	0.002	2.40E-09	0.002	0.008	8.05E-01
rs806795	6	26205293	A	G	0.470	360895	8.93E-05	32.230	Using the computer	0.010	0.002	1.40E-08	-0.005	0.008	5.00E-01
rs9375188	6	98555272	T	C	0.484	360895	2.07E-04	74.896	Using the computer	0.016	0.002	5.00E-18	-0.006	0.008	4.69E-01

rs9537571	13	57604700	A	G	0.096	360895	1.03E-04	37.220	Using the computer	0.019	0.003	1.10E-09	0.004	0.013	7.70E-01
rs11057408	12	124464836	T	G	0.334	310555	1.05E-04	32.529	Driving	-0.011	0.002	1.20E-08	-0.029	0.008	1.15E-04
rs12069474	1	98438135	C	A	0.219	310555	9.78E-05	30.381	Driving	0.012	0.002	3.50E-08	0.032	0.009	7.49E-04
rs12921753	16	30018720	T	C	0.400	310555	1.28E-04	39.761	Driving	0.012	0.002	2.90E-10	0.039	0.008	1.43E-06
rs2090660	2	136818719	T	C	0.194	310555	9.76E-05	30.311	Driving	-0.013	0.002	3.70E-08	0.015	0.010	1.28E-01
rs2588917	10	63524979	A	C	0.448	310555	9.81E-05	30.477	Driving	-0.010	0.002	3.40E-08	0.002	0.007	7.77E-01
rs975303	6	19028788	G	A	0.181	310555	1.18E-04	36.757	Driving	0.015	0.002	1.30E-09	-0.003	0.009	7.48E-01
rs10822175	10	65255174	T	A	0.882	343827	8.84E-05	30.394	Moderate physical activity	-0.019	0.003	3.50E-08	0.012	0.011	2.54E-01
rs1538360	6	98276688	G	A	0.553	343827	1.01E-04	34.726	Moderate physical activity	-0.012	0.002	3.80E-09	0.014	0.008	7.20E-02
rs682245	18	53270531	C	T	0.524	343827	1.04E-04	35.744	Moderate physical activity	0.012	0.002	2.30E-09	-0.019	0.008	1.45E-02
rs7222403	17	19879164	C	T	0.426	343827	8.83E-05	30.378	Moderate physical activity	0.011	0.002	3.60E-08	-0.012	0.007	9.15E-02
rs7610133	3	3880784	C	T	0.259	343827	8.67E-05	29.824	Moderate physical activity	-0.013	0.002	4.70E-08	0.021	0.009	1.68E-02
rs1248860	3	85015779	A	G	0.516	261055	2.12E-04	55.256	Vigorous physical activity	0.010	0.001	1.10E-13	-0.002	0.008	8.41E-01
rs13243553	7	133506955	A	G	0.392	261055	1.61E-04	42.020	Vigorous physical activity	-0.009	0.001	9.00E-11	0.012	0.008	1.20E-01
rs2764261	6	108927842	G	A	0.626	261055	1.72E-04	44.995	Vigorous physical activity	-0.009	0.001	2.00E-11	0.012	0.008	1.52E-01
rs328902	7	35020843	T	C	0.315	261055	1.47E-04	38.478	Vigorous physical activity	0.009	0.001	5.50E-10	-0.009	0.008	2.64E-01
rs3781411	10	126715436	T	C	0.124	261055	1.52E-04	39.669	Vigorous physical activity	-0.013	0.002	3.00E-10	0.023	0.011	3.28E-02
rs6667222	1	154253661	C	A	0.252	261055	1.27E-04	33.104	Vigorous physical activity	-0.009	0.002	8.70E-09	0.016	0.009	8.94E-02
rs9276758	6	32772975	A	G	0.312	261055	1.23E-04	32.126	Vigorous physical activity	-0.008	0.001	1.40E-08	0.017	0.009	5.42E-02
rs11012732	10	21830104	G	A	0.332	91084	3.74E-04	34.036	Accelerometer-based physical activity	-0.225	0.039	5.40E-09	-0.002	0.008	7.90E-01
rs12522261	5	152054825	A	G	0.343	91084	3.32E-04	30.207	Accelerometer-based physical activity	-0.211	0.038	3.90E-08	-0.017	0.008	3.93E-02
rs56194509	17	43844559	G	T	0.220	91084	5.23E-04	47.676	Accelerometer-based physical activity	0.303	0.044	5.00E-12	0.010	0.009	3.01E-01
rs59499656	18	40768309	T	A	0.344	91084	3.91E-04	35.597	Accelerometer-based physical activity	0.228	0.038	2.40E-09	-0.018	0.008	3.06E-02
rs6775319	3	18758501	T	A	0.729	91084	3.34E-04	30.430	Accelerometer-based physical activity	-0.225	0.041	3.50E-08	0.027	0.009	2.79E-03
rs9293503	5	87948962	C	T	0.112	91084	3.45E-04	31.420	Accelerometer-based physical activity	-0.329	0.059	2.10E-08	-0.024	0.013	5.92E-02

chr: chromosome; pos: position; EA: effect allele; OA: other allele; EAF: effect allele frequency.

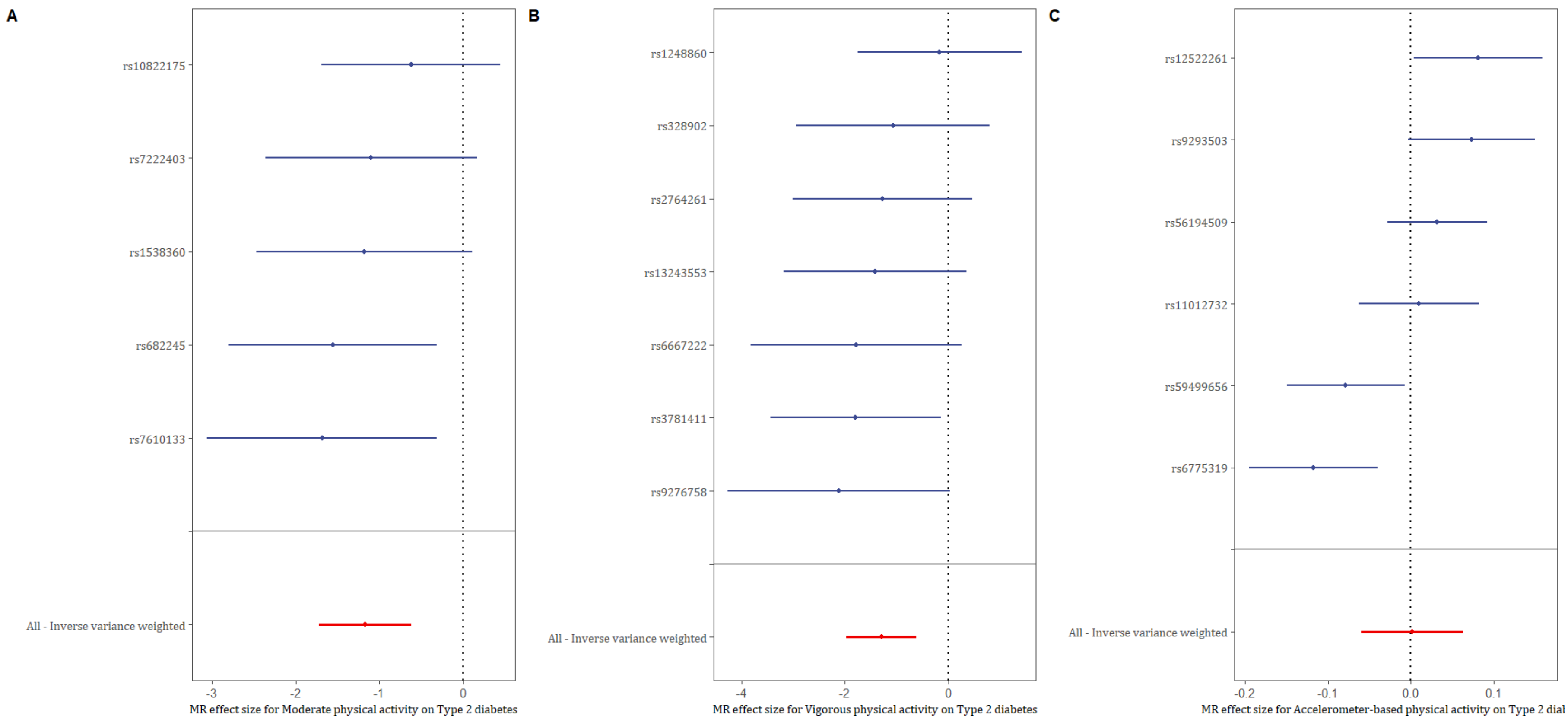

Supplementary Figure S1 Mendelian Randomization analysis for individual SNPs associated with physical activity in relation to type 2 diabetes risk

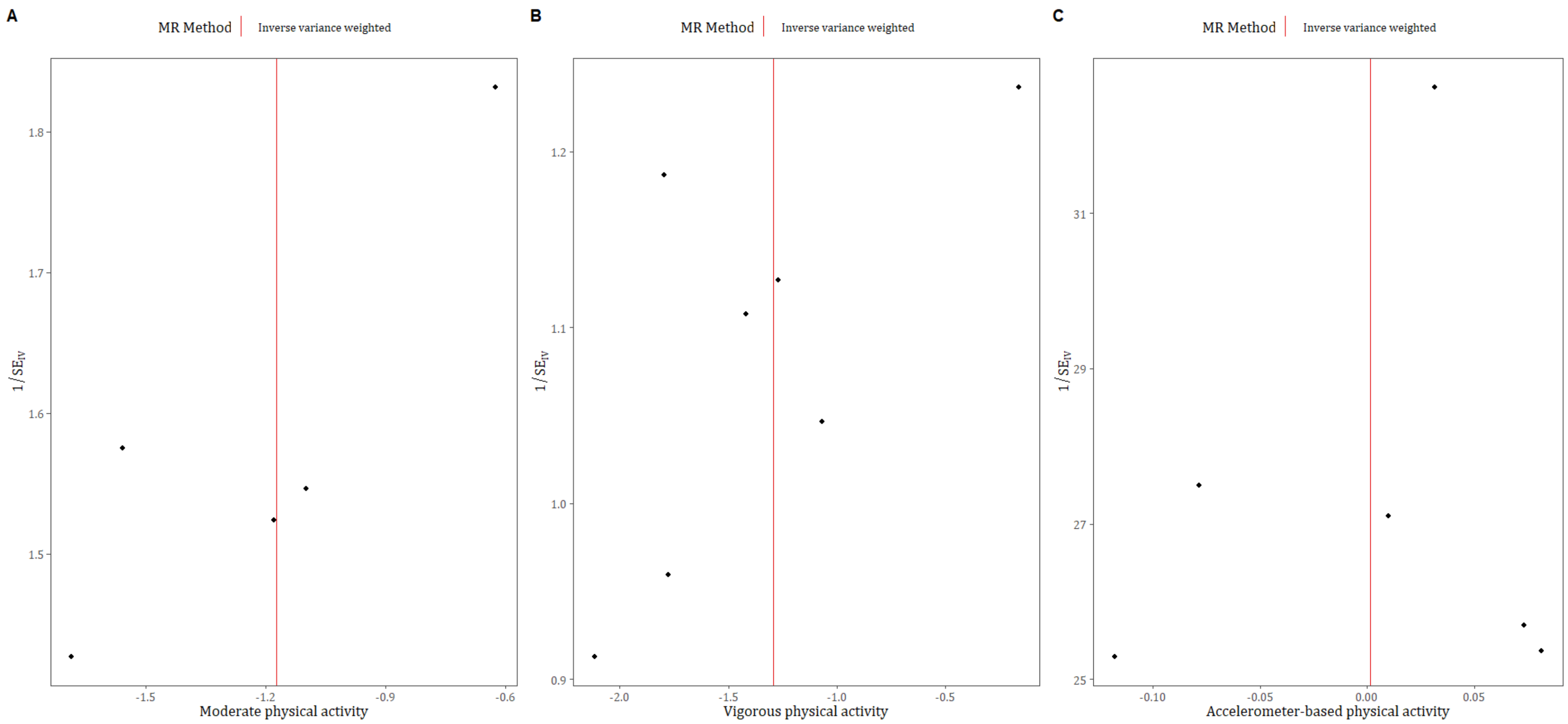

Supplementary Figure S2 Funnel plots of risk estimates of physical activity and type 2 diabetes

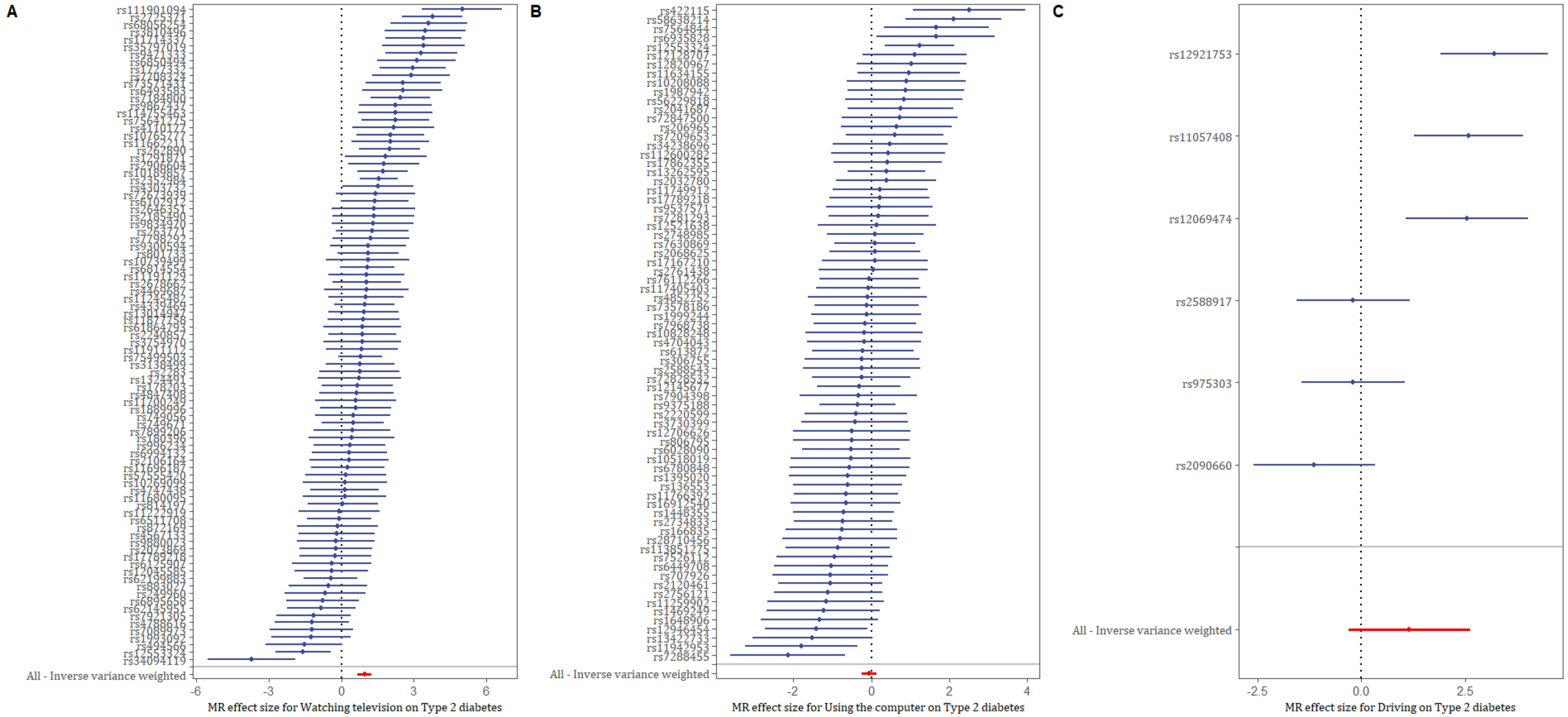

Supplementary Figure S3 Mendelian Randomization analysis for individual SNPs associated with sedentary behavior in relation to type 2 diabetes risk

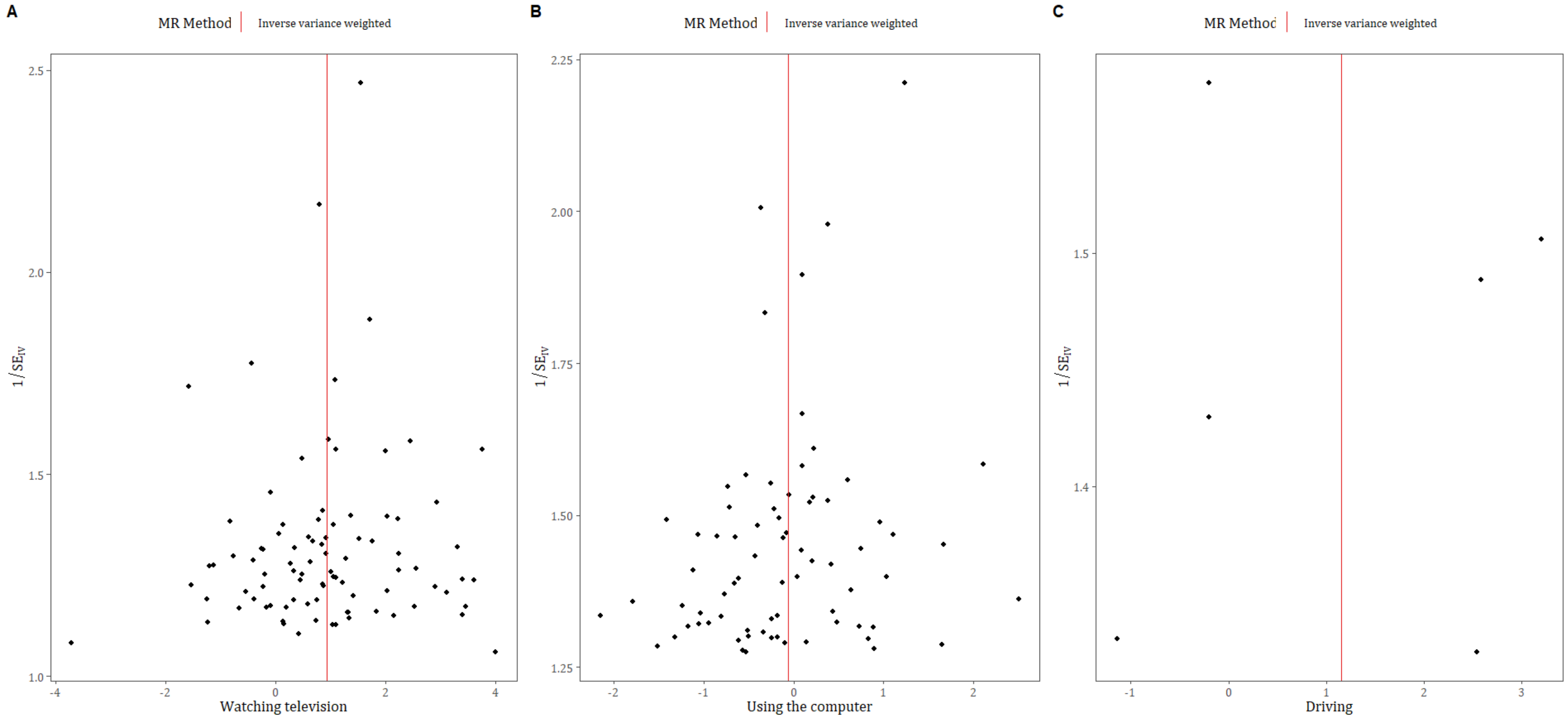

Supplementary Figure S4 Funnel plots of risk estimates of sedentary behavior and type 2 diabetes

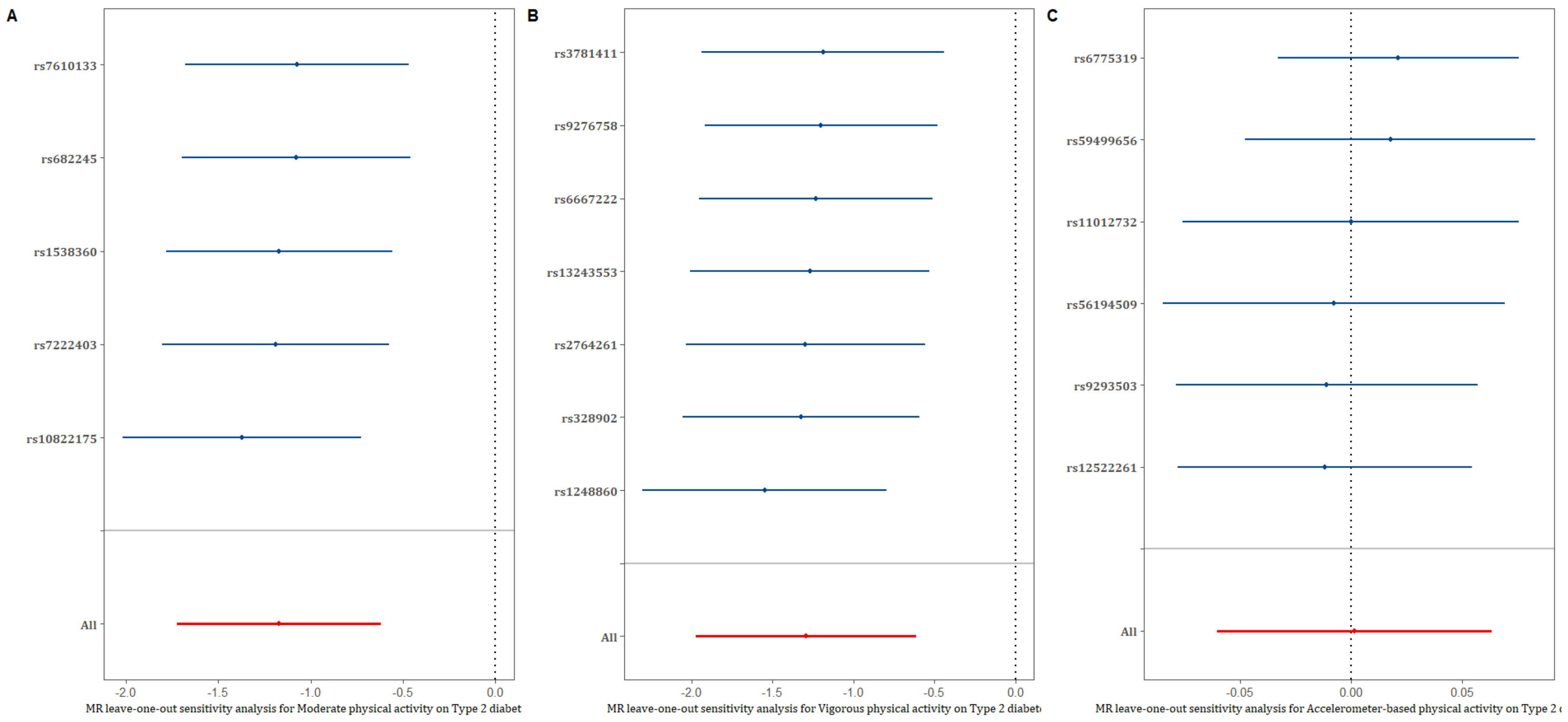

Supplementary Figure S5 Mendelian randomization estimates between physical activity and type 2 diabetes risk by leaving one SNP out at a time

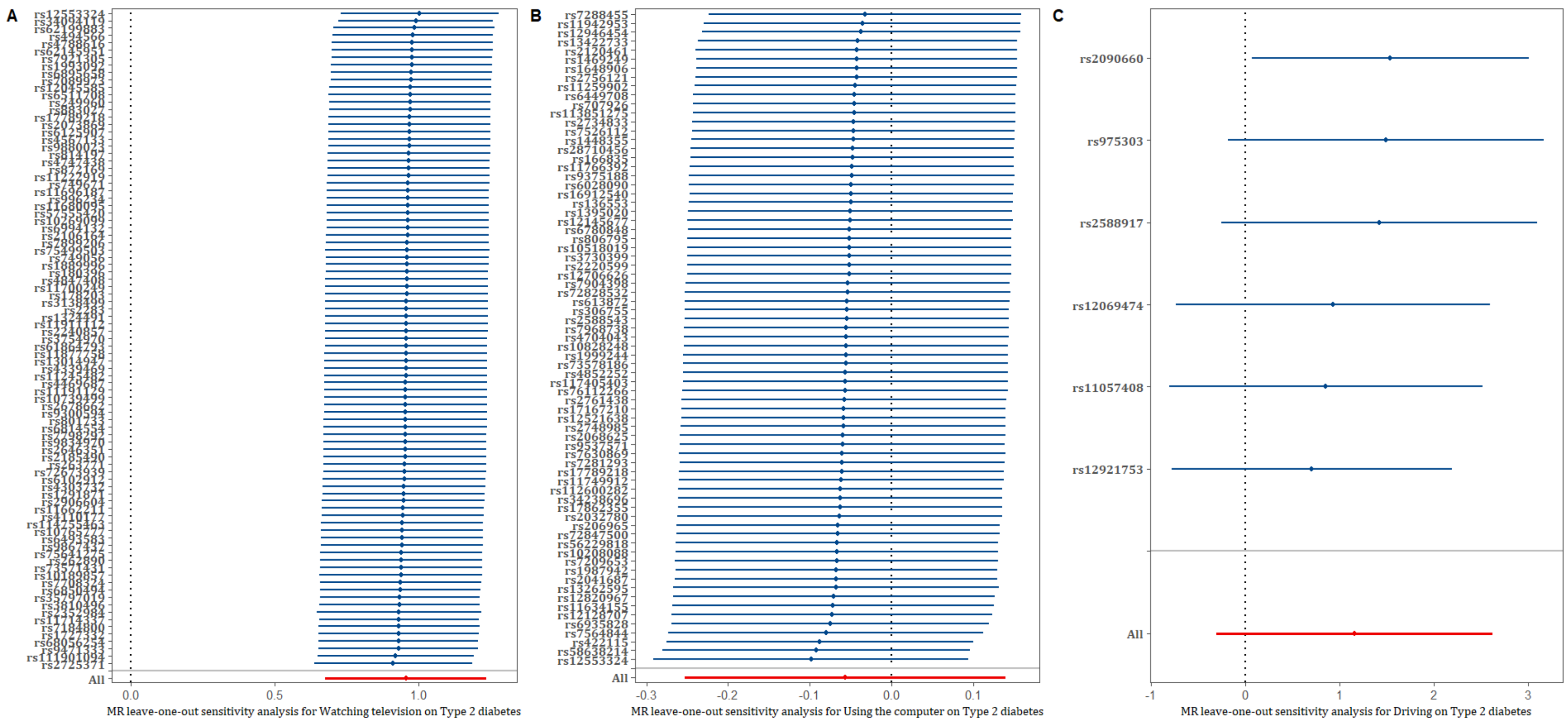

Supplementary Figure S6 Mendelian randomization estimates between sedentary behaviors and type 2 diabetes risk by leaving one SNP out at a time
